# Current and Former Smoking and Risk for Venous Thromboembolism: A Systematic Review and Meta-Analysis

**DOI:** 10.1371/journal.pmed.1001515

**Published:** 2013-09-17

**Authors:** Yun-Jiu Cheng, Zhi-Hao Liu, Feng-Juan Yao, Wu-Tao Zeng, Dong-Dan Zheng, Yu-Gang Dong, Su-Hua Wu

**Affiliations:** 1 Department of Cardiology, First Affiliated Hospital, Sun Yat-Sen University, Guangzhou, China; 2 Department of Gastroenterology, the Third Affiliated Hospital, Sun Yat-Sen University, Guangzhou, China; 3 Department of Ultrasonography, the First Affiliated Hospital, Sun Yat-Sen University, Guangzhou, China; University of Glasgow, United Kingdom

## Abstract

**Background:**

Smoking is a well-established risk factor for atherosclerotic disease, but its role as an independent risk factor for venous thromboembolism (VTE) remains controversial. We conducted a meta-analysis to summarize all published prospective studies and case-control studies to update the risk for VTE in smokers and determine whether a dose–response relationship exists.

**Methods and Findings:**

We performed a literature search using MEDLINE (source PubMed, January 1, 1966 to June 15, 2013) and EMBASE (January 1, 1980 to June 15, 2013) with no restrictions. Pooled effect estimates were obtained by using random-effects meta-analysis. Thirty-two observational studies involving 3,966,184 participants and 35,151 VTE events were identified. Compared with never smokers, the overall combined relative risks (RRs) for developing VTE were 1.17 (95% CI 1.09–1.25) for ever smokers, 1.23 (95% CI 1.14–1.33) for current smokers, and 1.10 (95% CI 1.03–1.17) for former smokers, respectively. The risk increased by 10.2% (95% CI 8.6%–11.8%) for every additional ten cigarettes per day smoked or by 6.1% (95% CI 3.8%–8.5%) for every additional ten pack-years. Analysis of 13 studies adjusted for body mass index (BMI) yielded a relatively higher RR (1.30; 95% CI 1.24–1.37) for current smokers. The population attributable fractions of VTE were 8.7% (95% CI 4.8%–12.3%) for ever smoking, 5.8% (95% CI 3.6%–8.2%) for current smoking, and 2.7% (95% CI 0.8%–4.5%) for former smoking. Smoking was associated with an absolute risk increase of 24.3 (95% CI 15.4–26.7) cases per 100,000 person-years.

**Conclusions:**

Cigarette smoking is associated with a slightly increased risk for VTE. BMI appears to be a confounding factor in the risk estimates. The relationship between VTE and smoking has clinical relevance with respect to individual screening, risk factor modification, and the primary and secondary prevention of VTE.

*Please see later in the article for the Editors' Summary*

## Abstract 2

In a meta-analysis of 32 observational studies involving 3,966,184 participants and 35,151 events, Suhua Wu and colleagues found that current, ever, and former smoking was associated with risk of venous thromboembolism.

*Please see later in the article for the Editors' Summary*

## Editors' Summary

**Background:**

Blood normally flows throughout the human body, supplying its organs and tissues with oxygen and nutrients. But, when an injury occurs, proteins called clotting factors make the blood gel (coagulate) at the injury site. The resultant clot (thrombus) plugs the wound and prevents blood loss. Occasionally, a thrombus forms inside an uninjured blood vessel and partly or completely blocks the blood flow. Clot formation inside one of the veins deep within the body, usually in a leg, is called deep vein thrombosis (DVT) and can cause pain, swelling, and redness in the affected limb. DVT can be treated with drugs that stop the blood clot from getting larger (anticoagulants) but, if left untreated, part of the clot can break off and travel to the lungs, where it can cause a life-threatening pulmonary embolism. DVT and pulmonary embolism are collectively known as venous thromboembolism (VTE). Risk factors for VTE include having an inherited blood clotting disorder, oral contraceptive use, prolonged inactivity (for example, during a long-haul plane flight), and having surgery. VTEs are present in about a third of all people who die in hospital and, in non-bedridden populations, about 10% of people die within 28 days of a first VTE event.

**Why Was This Study Done?:**

Some but not all studies have reported that smoking is also a risk factor for VTE. A clear demonstration of a significant association (a relationship unlikely to have occurred by chance) between smoking and VTE might help to reduce the burden of VTE because smoking can potentially be reduced by encouraging individuals to quit smoking and through taxation policies and other measures designed to reduce tobacco consumption. In this systematic review and meta-analysis, the researchers examine the link between smoking and the risk of VTE in the general population and investigate whether heavy smokers have a higher risk of VTE than light smokers. A systematic review uses predefined criteria to identify all the research on a given topic; meta-analysis is a statistical method for combining the results of several studies.

**What Did the Researchers Do and Find?:**

The researchers identified 32 observational studies (investigations that record a population's baseline characteristics and subsequent disease development) that provided data on smoking and VTE. Together, the studies involved nearly 4 million participants and recorded 35,151 VTE events. Compared with never smokers, ever smokers (current and former smokers combined) had a relative risk (RR) of developing VTE of 1.17. That is, ever smokers were 17% more likely to develop VTE than never smokers. For current smokers and former smokers, RRs were 1.23 and 1.10, respectively. Analysis of only studies that adjusted for body mass index (a measure of body fat and a known risk factor for conditions that affect the heart and circulation) yielded a slightly higher RR (1.30) for current smokers compared with never smokers. For ever smokers, the population attributable fraction (the proportional reduction in VTE that would accrue in the population if no one smoked) was 8.7%. Notably, the risk of VTE increased by 10.2% for every additional ten cigarettes smoked per day and by 6.1% for every additional ten pack-years. Thus, an individual who smoked one pack of cigarettes per day for 40 years had a 26.7% higher risk of developing VTE than someone who had never smoked. Finally, smoking was associated with an absolute risk increase of 24.3 cases of VTE per 100,000 person-years.

**What Do These Findings Mean?:**

These findings indicate that cigarette smoking is associated with a statistically significant, slightly increased risk for VTE among the general population and reveal a dose-relationship between smoking and VTE risk. They cannot prove that smoking causes VTE—people who smoke may share other unknown characteristics (confounding factors) that are actually responsible for their increased risk of VTE. Indeed, these findings identify body mass index as a potential confounding factor that might affect the accuracy of estimates of the association between smoking and VTE risk. Although the risk of VTE associated with smoking is smaller than the risk associated with some well-established VTE risk factors, smoking is more common (globally, there are 1.1 billion smokers) and may act synergistically with some of these risk factors. Thus, smoking behavior should be considered when screening individuals for VTE and in the prevention of first and subsequent VTE events.

**Additional Information:**

Please access these Web sites via the online version of this summary at http://dx.doi.org/10.1371/journal.pmed.1001515.

- The US National Heart Lung and Blood Institute provides information on deep vein thrombosis (including an animation about how DVT causes pulmonary embolism), and information on pulmonary embolism
- The UK National Health Service Choices website has information on deep vein thrombosis, including personal stories, and on pulmonary embolism; SmokeFree is a website provided by the UK National Health Service that offers advice on quitting smoking
- The non-profit organization US National Blood Clot Alliance provides detailed information about deep vein thrombosis and pulmonary embolism for patients and professionals and includes a selection of personal stories about these conditions
- The World Health Organization provides information about the dangers of tobacco (in several languages)
- Smokefree.gov, from the US National Cancer Institute, offers online tools and resources to help people quit smoking
- MedlinePlus has links to further information about deep vein thrombosis, pulmonary embolism, and the dangers of smoking (in English and Spanish)

## Introduction

Although cigarette smoking has been responsible for approximately 5 million deaths every year, there are still an estimated 1.1 billion smokers worldwide [1],[2]. The magnitude of this public health challenge is growing, and estimates suggest that as many as 8 million people may die from smoking-related diseases by 2030 [2]. Venous thromboembolism (VTE) is a serious medical event and associated with a substantial risk of mortality [3]. In ambulatory population-based cohorts, the estimated 28-d mortality for the first episode of VTE is 11% [4]. Autopsy studies have found that VTE exists in about one-third of deaths in hospitals and 13% of all autopsies showed signs of fatal pulmonary embolism (PE) [5],[6].

Smoking is a well-established risk factor for atherosclerotic disease, but its role as an independent risk factor or effect modifier for VTE remains controversial. Several prospective studies reported smoking to be an independent risk factor [7],[8], whereas others failed to detect a significant relationship between smoking and VTE [9],[10]. A recent meta-analysis showed a statistically nonsignificant odds ratio (OR) for VTE of 1.18 (95% CI 0.95–1.46) for smokers compared with non-smokers [11]. However, the meta-analysis (involving a total of ten studies) included only about one-third of the data currently available. In addition, six of the ten studies included were clinical trials of oral contraceptives, in which the samples may not be representative of the general population. Furthermore, the VTE risk may be underestimated due to lack of distinction between former and current smokers and no adjustment for cardiovascular risk factors.

Smoking can be potentially reduced by individual and population-related measures; therefore, demonstrating the link between smoking and the risk of VTE may help reduce the burden of this disease. Therefore, we conducted a meta-analysis with the following aims: (1) to estimate the link between smoking and risk of VTE in the general population; (2) to measure the smoking-VTE relationship according to different degrees of adjustment for confounding factors, study designs, study populations, sex category, and type of VTE; and (3) to study dose-response patterns of tobacco exposure on the risk of VTE.

## Methods

### Search Strategy

This meta-analysis follows PRISMA guidelines (Text S1). We searched the publications listed in the electronic databases MEDLINE (source PubMed, January 1, 1966 to June 15, 2013) and EMBASE (January 1, 1980 to June 15, 2013) using the following text and key words in combination both as MeSH terms and text word “thromboembolism”, “venous thrombosis”, “pulmonary embolism”, deep-vein thrombosis”, “risk factors”, “smoke”, “cigarette”, “tobacco” or “smoking”. We searched articles published in any language and scrutinized references from these studies to identify other relevant studies.

### Study Selection

To minimize differences between studies, we imposed the following methodological restrictions for the inclusion criteria: (1) Studies that contained the minimum information necessary to estimate the relative risk (RR) associated with smoking, including case-control and cohort studies published as original articles; (2) Studies in which populations were representative of the general population and not those with selected participants on the basis of risk factor for VTE, such as tumor, surgery, or use of oral contraceptives. In instances of multiple publications, the most up-to-date or comprehensive information was used.

### Data Abstraction

Articles were reviewed and cross-checked independently by two authors (YJC and ZHL). Because there is no standardized quality scoring system for observational studies, we selected a priori several important design characteristics that may affect study quality, including method of case confirmation, percentage of patients completing planned follow-up, smoking as the primary analysis of interest, selection criteria for control participants, matching criteria, and control for confounding. Percentage agreement between the two authors on the quality review ranged from 88% to 100%. Any disagreements were resolved by consensus. Data on the following characteristics were independently extracted: study size, number of patients who developed VTE, total person-years of follow-up, study population, publication year, study design, sampling framework, study location (defined as Europe, North America, or Asia); gender category, site of VTE studied (deep vein thrombosis [DVT] or PE), type of VTE studied (unprovoked or provoked), ascertainment of VTE (validated or not validated), smoking category (ever, current, or former), and reported adjustment for potential confounders. When available, we used the most comprehensively adjusted risk estimates.

### Data Analysis

RR was used as a measure of the relationship between smoking and the risk of VTE. For case-control studies, the OR was used as a surrogate measure of the corresponding RR. Because the absolute risk of VTE is low, the OR approximates the RR [12].

Summary RRs (95% CI) were calculated by pooling the study-specific estimates using a random-effects model that included between-study heterogeneity (parallel analyses used fixed-effects models), because significant heterogeneity was anticipated among studies. Pooled RRs were expressed with 95% CIs. We calculated the I^2^ (95% CI) statistic to assess heterogeneity across studies, applying the following interpretation for I^2^: <50% = low heterogeneity; 50%–75% = moderate heterogeneity; >75% = high heterogeneity [13].

We calculated the population attributable fraction (PAF) as {prevalence of smoking×(RR−1)/[prevalence of smoking×(RR−1)+1]}, where RR indicates pooled RRs [14]. On the basis of population-based cohort studies, the average prevalence of three categories of smoking was estimated by weighting by the sample size of each study.

Subgroup analyses and meta-regression models were carried out to investigate potential sources of between-study heterogeneity. When several risk estimates were present in a single study (i.e., separate estimates for current and former smokers), we adjusted the pooled estimates for intra-study or within-study correlation [15].

In the dose-response analysis, we considered cigarettes per day and pack-years as explanatory variables. Because for many studies continuous exposures were reported as categorical data with a range, we assigned the mid-point in each category to the corresponding RR for each study. When the highest category was open ended, we considered 60 cigarettes per day and 60 pack-years as the maximum (for example, one study reported >20 cigarettes per day as an open range; we considered 40 cigarettes per day as the mid-point in this category). We used generalized least squares (GLST) regression models to assess the pooled dose-response relation between smoking and risk of VTE across studies that had heterogeneous categorizations of smoking [16]. Linear models were fitted and evaluated on the logarithm of the RRs.

To enable the total person-years of observation to be calculated, we included data from reports that specified one or more of the following: total person-time of follow-up; sample size and mean (or median) follow-up per patient; or sample size and cumulative incidence rate. The principal summary measure was event rate expressed per 100,000 patient-years of follow-up. Weighted meta-analytic prevalence estimates for outcomes were calculated with the variance-stabilizing Freeman-Tukey double-arcsine transformation with an inverse-variance random-effects model [17].

Small study bias, consistent with publication bias, was assessed with funnel plot, by Begg's adjusted rank correlation test and by Egger's regression asymmetry test [18]. We used STATA, version 11.0 (Stata Corp) for all analyses. Statistical tests were two sided and used a significance level of *p*<0.05.

## Results

### Study Selection

**Figure 1:**
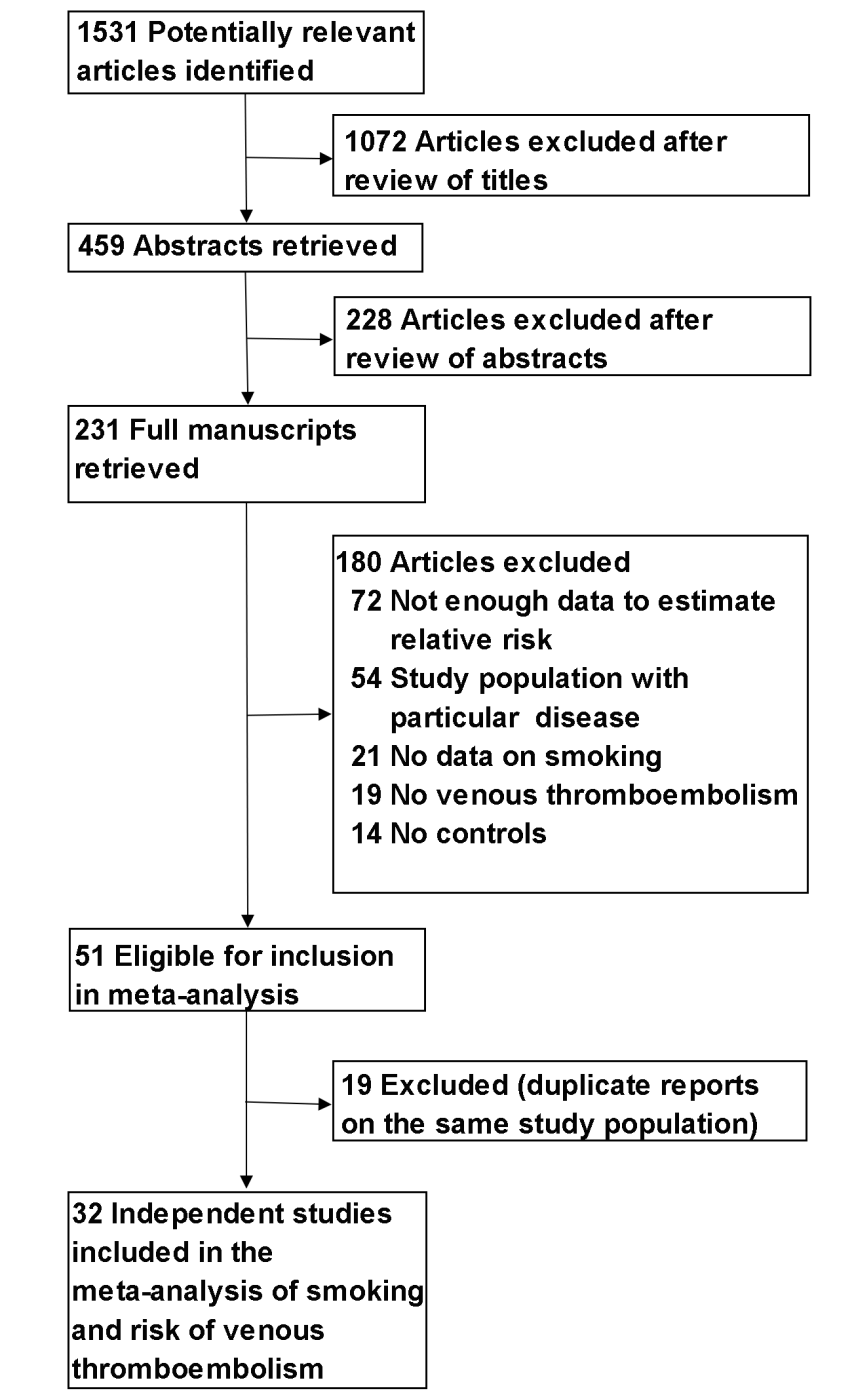
Flowchart of the selection of studies included in meta-analysis.

With the search strategy, 1,531 unique citations were initially retrieved. Of these, 231 articles were considered of interest and full text was retrieved for detailed evaluation. One hundred ninety-nine of these 231 articles were subsequently excluded and finally 32 articles were included in the meta-analysis (Figure 1).

### Study Characteristics

Thirty-two independent observational studies reporting 3,966,184 individuals and 35,151 incident cases were identified [7],[8],[19]–[48]. Fifteen studies were based in Europe, eight in North America, and nine in Asia. No studies were based in Africa or South America. Studies were published between January 1980 and March 2013. Thirteen studies were prospective cohort studies and 19 were case-control studies. 15 studies recruited participants from population registers and 15 were hospital-based.

The methodological quality of the included studies was generally good. Of the primary studies, 100% had described independent, consecutive sampling of their cohort. Average follow-up duration ranged from 5.0 to 20.1 y. Patients were followed up for an average of over 10 y in a majority of studies (84.6%). The proportion of patients with complete follow-up to the end of the study was given for 11 studies and ranged from 70.5% to >99%. The sizes of the cohorts ranged from 855 to 2,314,701 (in total 3,926,048), with the two largest studies recruiting participants over 1 million (Table 1) [26],[29]. Nineteen case-control studies were designed to evaluate risk factors for VTE, and eight of them used either hospital discharge data or data from registries. In 12 of the 19 incident case-control studies, controls were matched for age and/or sex only (Table 2).

**Table 1:** Cohort studies reporting incidence risk estimates.

Study	Year	Country	Source	Mean Follow-up (y)	Case Confirmation	Sex^a^	Female (%)	*n* Cases	Persons at Risk	Type of VTE	Site of VTE	Variables adjusted for^b^	Smoking Category
Goldhaber SZ [19]	1997	USA	Population-based	14.3	Questionnaire	W	100	280	112,822	Unprovoked or provoked	PE	Age, BMI, cholesterol, diabetes, hypertension, and other	Current, former
Hansson PO [20]	1999	Sweden	Population-based	13	Medical record and radiology	M	0	56	855	Unprovoked or provoked	DVT or PE	Waist circumference	Current, former
Klatsky AL [21]	2000	USA	Population-based	14.1	Radiology and autopsy	Both	51.3	337	128,934	Unprovoked or provoked	DVT or PE	Age, sex, BMI, alcohol, and other	Ever
Glynn RJ [22]	2005	USA	Clinical trial	20.1^c^	Questionnaire	M	0	358	18,662	Unprovoked, provoked	DVT or PE	Age, BMI, cholesterol, diabetes, hypertension, alcohol, physical activity, and other	Current, former
Lindqvist PG [23]	2008	Sweden	Population-based	11	Questionnaire	W	100	312	2,498	Unprovoked or provoked	DVT or PE	Age	Ever
Rosengren A [24]	2008	Sweden	Population-based	14.4	Medical record and radiology	M	0	358	6,958	Unprovoked or provoked	DVT or PE	Age	Current, former
Severinsen MT [8]	2009	Denmark	Population-based	10.2^c^	Medical record and radiology	M, W	52.3	641	57,053	Unprovoked, provoked	DVT or PE	BMI, alcohol, physical activity, and other	Current, former
Holst AG [7]	2010	Denmark	Population-based	19.5^c^	Death and patient registry	M, W	53.5	969	18,954	unprovoked	DVT or PE,	Sex, BMI, blood pressure, and other	Current, former
Lutsey PL [25]	2010	USA	Population-based	13^c^	Questionnaire	W	100	2,137	40,377	Unprovoked or provoked	DVT or PE	Age, BMI, physical activity, and other	Current, former
Hippisley-Cox J [26]	2011	UK	General practitioner register	5	Death and patient registry	M, W	48.6	14,756	2,314,701	Unprovoked or provoked	DVT or PE	Age, BMI, and other	Current, former
Enga KF [27]	2012	Norway	Population-based	12.5^c^	Medical record and radiology or autopsy	Both	53.3	389	24,576	Unprovoked, provoked	DVT or PE	Age, sex, BMI, and other	Current, former
Wattanakit K [28]	2012	USA	Population-based	15.5	Medical record and radiology or autopsy	Both	55.4	468	15,340	Unprovoked, provoked	DVT or PE	Age, sex, BMI, and other	Current, former
Sweetland S [29]	2013	UK	Population-based	6	Questionnaire	W	100	4,630	1,162,718	Unprovoked or provoked	DVT, PE	Age, BMI, diabetes, hypertension, alcohol, physical activity, and other	Current, former

BMI calculated as weight in kilograms divided by height in meters squared.

a Adjusted estimates were reported for men and women separately and together. If all three types of estimates were reported (M, W, B), they were analyzed separately by sex only in the heterogeneity analysis for sex.

b The term “other” in the “Variables adjusted for” column stands for all the adjusting variables other than age, sex, and cardiovascular risk factors (BCDHAP: B, BMI, body weight, waist circumference; C, cholesterol; D, diabetes; H, hypertension; A, alcohol consumption; P, physical activity).

c Median.

**Table 2:** Case-control studies reporting incidence risk estimates.

Study	Year	Country	Source	Control Group	Case Confirmation	Sex^a^	Female (%)	*n* Cases	*n* Controls	Type of VTE	Site of VTE	Variables Adjusted for^b^	Smoking Category
Dreyer NA [30]	1980	USA	Hospital-based	Age and race matched	Radiology	W	100	15	29	Unprovoked	DVT or PE	None	Ever
Lu Y [31]	2001	China	Hospital-based	Sex and age matched	Radiology	Both	38.9	72	72	Unprovoked or provoked	PE	None	Ever
Ray JG [32]	2001	Canada	Hospital-based	Age matched	Radiology	W	100	129	129	Unprovoked or provoked	DVT or PE	None	Current
Tosetto A [33]	2003	Italy	Population-based	Asymptomatic individuals	Questionnaire	Both	53.2	116	14,939	Unprovoked, provoked	DVT, PE,	Age, sex, BMI, and other	Ever
Worralurt C [34]	2005	Thailand	Hospital-based	Age and education matched	Radiology	W	100	70	140	Unprovoked or provoked	DVT or PE	None	Ever
Hirohashi T [35]	2006	Japan	Hospital-based	Non-VTE patients	Radiology	Both	46.9	75	151	Unprovoked or provoked	PE	None	Ever
Sugimura K [36]	2006	Japan	Hospital-based	Sex and age matched	Questionnaire	Both	67	209	209	Unprovoked or provoked	DVT	None	Ever
Pomp ER [37]	2007	The Netherlands	Population-based	Partner matched	Radiology	M,W	100	3,989	4,900	Unprovoked or provoked	DVT, PE	Age, sex, BMI and other	Current, former
Prandoni P [38]	2008	Italy	Hospital-based	Sex and age matched	Radiology	Both	54.6	299	150	Unprovoked, provoked	DVT or PE	None	Ever
Jang MJ [39]	2009	Korea	Hospital-based	Healthy individuals	Objectively diagnosed	Both	57.1	208	300	Unprovoked, provoked	DVT or PE	Age, sex, BMI, hypertension, cholesterol, glucose	Ever
Yamada N [40]	2009	Japan	Hospital-based	Non-VTE patients	Radiology	Both	47.8	100	199	Unprovoked or provoked	PE	Age, sex	Ever
Bhoopat L [41]	2010	Tailand	Hospital-based	Sex and age matched	Radiology	Both	69.7	97	195	Unprovoked or provoked	DVT or PE	None	Ever
Quist-Paulsen P [42]	2010	Norway	Population-based	Sex and age matched	Medical record and radiology	Both	54.6	483	1,362	Unprovoked or provoked	DVT or PE	Age, sex	Ever
Zhu J [43]	2010	China	Hospital-based	Sex and age matched	Patients hospitalized	Both	48.8	425	527	Unprovoked or provoked	DVT or PE	Age, sex, body weight, and other	Current, former
Cay N [44]	2011	Turkey	Hospital-based	Non-VTE patients	Radiology	Both	43.3	203	210	Unprovoked or provoked	DVT	None	Ever
Di Minno MN [45]	2010	Italy	Hospital-based	Sex and age matched	Radiology	M, W, Both	63.6	323	868	Unprovoked	DVT or PE	None	Ever
Abudureheman K [46]	2012	China	Hospital-based	Healthy individuals	Radiology	Both	49.8	222	220	Unprovoked or provoked	DVT or PE	Age, sex, BMI, cholesterol, hypertension, glucose, and other	Ever
Cil H [47]	2012	Turkey	Hospital-based	Healthy individuals	Medical record and radiology	Both	50.1	147	149	Unprovoked or provoked	DVT or PE	Age, BMI, hypertension, and other	Ever
Blondon M [48]	2013	USA	Population-based	Age matched	Medical record and radiology	W	54.6	2,278	5,927	Unprovoked, provoked	DVT or PE,	Age, BMI, hypertension, diabetes, and other	Current, former

BMI, calculated as weight in kilograms divided by height in meters squared.

a Adjusted estimates were reported for men and women separately and together. If all three types of estimates were reported (M, W, B), they were analyzed separately by sex only in the heterogeneity analysis for sex.

b The term “other” in the “Variables adjusted for” column stands for all the adjusting variables other than age, sex and cardiovascular risk factors (BCDHAP: B, BMI, body weight, waist circumference; C, cholesterol; D, diabetes; H, hypertension; A, alcohol consumption; P, physical activity).

Of all the studies, two included only patients with DVT [36],[44] and four investigated only patients with PE [19],[31],[35],[40]. Four cohort studies [8],[22],[27],[28] and four case-control studies [33],[38],[39],[48] compared the prevalence of smoking between patients with unprovoked VTE and provoked VTE. Eight studies investigated only women [19],[23],[25],[29],[30],[32],[34],[48] and three studies included only men [20],[22],[24]. The association between smoking and VTE was the primary outcome of interest for 20 studies, whereas it was a secondary question in 12 studies. The ascertainment of VTE varied across studies; 24 studies based on medical record, radiology or autopsy (validated), and eight confirmed by questionnaire or patient registry (not validated) (Tables 1 and 2).

Adjusted RRs could be determined for all cohort studies and nine of the case-control studies. Most risk estimates were adjusted for age (19 studies) and sex (11 studies). Eighteen studies (56.3%) reported an adjusted estimate for at least one of the cardiovascular risk factors: BMI (11 cohort and seven case-control studies), cholesterol (three cohort and one case-control studies), diabetes (three cohort and three case-control studies), hypertension (four cohort and four case-control studies), alcohol consumption (four cohort studies), or physical activity (four cohort studies). Detailed information on adjustments is reported in Tables 1 and 2.

### Smoking and Risk of VTE

**Figure 2:**
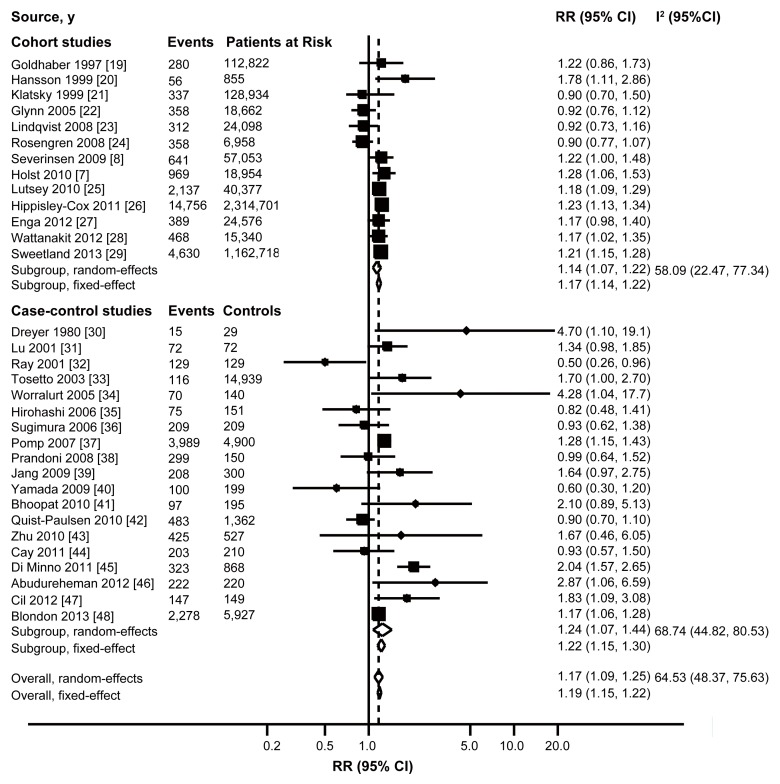
Forest plot for VTE incidence: risk estimates for ever versus never smokers. The size of each square is proportional to the study's weight (inverse of variance).

Figures 2, S1, and S2 showed the results from the random-effects model (parallel analysis with fixed-effects model) combining the RRs for VTE. Overall, the ever smokers compared with the reference group experienced a significantly increased risk for developing VTE (RR: 1.17 [95% CI 1.09–1.25, *p*<0.001]). The pooled RRs for current versus never smokers and former versus never smokers were 1.23 (95% CI 1.14–1.33, *p*<0.001) and 1.10 (95% CI 1.03–1.17, *p* = 0.002), respectively.

There was evidence of moderate heterogeneity of RRs across these studies. The findings from the sensitivity analyses based on different inclusion and exclusion criteria were presented in Table 3. Risk estimates changed little after analyses with fixed effects models, inclusion of the studies with adjusted RRs, or exclusion of the two largest and the outlier studies, yet moderate heterogeneity was still present. However, when the analysis was confined to those large prospective cohort studies (high quality), the overall combined RR did not materially change, but heterogeneity was decreased to 34.68% for ever smokers, 10.61% for current smokers, and 0% for former smokers.

**Table 3:** Sensitivity and heterogeneity analysis of pooled relative risks of VTE for smokers.

	Ever Versus Never Smoker				Current Versus Never Smoker				Former Versus Never Smoker			
	*n* Studies	RR (95% CI)	I^2^ (95% CI)	*p*-Value^a^	*n* Studies	RR (95% CI)	I^2^ (95% CI)	*p*-Value^a^	*n* Studies	RR (95% CI)	I^2^ (95% CI)	*p*-Value^a^
**Statistical model**												
Random effects	32	1.17 (1.09–1.25)	64.53 (48.37–75.63)	<0.001	15	1.23 (1.14–1.33)	64.89 (31.17–79.73)	<0.001	14	1.10 (1.03–1.17)	53.52 (14.82–74.64)	0.009
Fixed effects	32	1.19 (1.15–1.22)			15	1.29 (1.24–1.34)			14	1.09 (1.06–1.12)		
**Analysis of all studies with**												
Adjusted risk estimate^b^	22	1.16 (1.09–1.23)	58.27 (33.09–73.97)	0.01	14	1.25 (1.17–1.35)	59.04 (26.04–77.29)	0.003	14	1.10 (1.03–1.17)	53.52 (14.82–74.64)	0.009
Large cohort^c^	11	1.17 (1.11–1.23)	34.68 (0.00–67.91)	0.12	9	1.32 (1.26–1.38)	10.61 (0.00–68.53)	0.35	9	1.07 (1.04–1.11)	0.00 (0.00–64.80)	0.52
**Analysis of all studies except**												
Two largest studies^d^	30	1.17 (1.07–1.27)	65.99 (50.08–76.83)	<0.001	13	1.19 (1.07–1.33)	64.94 (36.72–80.58)	0.001	12	1.10 (1.01–1.22)	57.90 (20.10–77.82)	0.006
One outlier study^e^	31	1.17 (1.09–1.25)	64.21 (47.54–75.58)	<0.001	12	1.23 (1.13–1.33)	66.18 (40.56–80.76)	<0.001	12	1.08 (1.05–1.12)	0.00 (0.00–56.59)	0.51

a *p*-Value for I^2^.

b Studies reporting estimates that adjusted for at least one confounding factor.

c Large prospective cohort studies with sample size over 15,000.

d Studies with population over 1 million by Hippisley-Cox J and Sweetland S [26],[29].

e Studies with largest RR by Dreyer NA for ever smokers, by Hansson PO for current smokers and by Zhu J for former smokers [20],[30],[43].

**Figure 3:**
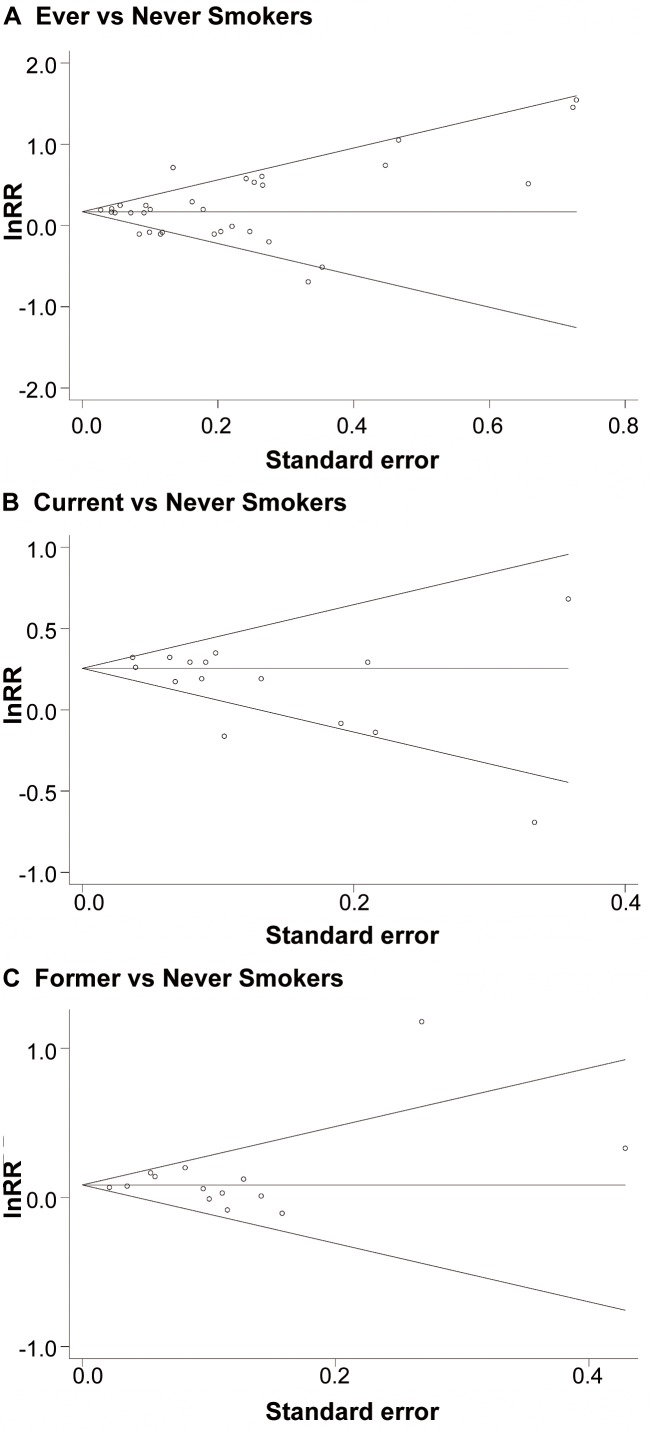
(A–C) Funnel plots showing associations of smoking with VTE.

Because the study by Ray et al. [32] only reported risk estimates for current but not for former smokers, in order to allow an unbiased comparison between the two smoking classes, we also computed the RR for current smokers (RR: 1.25 [95% CI 1.17–1.35]) from the remaining 14 studies reporting both estimates. Compared with former smokers, current smokers experienced a significant higher risk for developing VTE (*p* = 0.02). Neither funnel plots nor Egger and Begg tests showed evidence of publication bias for ever smokers (Egger, *p* = 0.88; Begg, *p* = 0.21), current smokers (Egger, *p* = 0.06; Begg, p = 0.11), and former smokers (Egger, *p* = 0.41; Begg, *p* = 0.83) (Figure 3).

### Stratified Analyses

**Figure 4:**
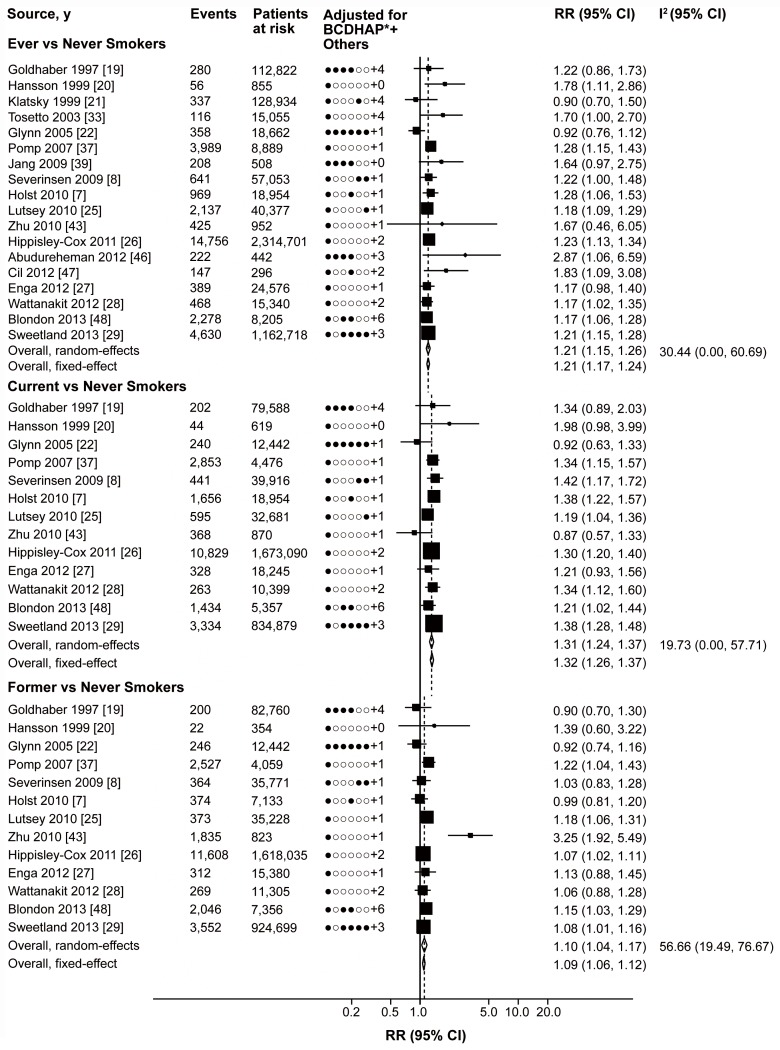
Cardiovascular risk factor-adjusted relative risk for VTE. The size of each square is proportional to the study's weight (inverse of variance). Cardiovascular risk factors (BCDHAP): B, BMI, body weight or waist circumference; C, cholesterol; D, diabetes; H, hypertension; A, alcohol consumption; P, physical activity. For example, Sweetland S 2013 adjusted for body mass index, diabetes, hypertension, alcohol consumption, physical activity, and three other non-cardiovascular risk factors, but not for cholesterol.

To explore study heterogeneity, we performed stratified analyses across a number of key study characteristics and clinical factors (Table 4). The finding of increased VTE risk in smokers was consistently observed in most of the stratified analyses. Study design, geographical area, or publication year were not significant sources of heterogeneity. In addition, the RRs in studies in which VTE cases were validated with imaging examination or medical record were not systematically different from studies in which they were not (Table 4; Figures S3, S4, S5). Level of adjustment in the primary studies seemed to be associated with the results (*p* = 0.03 for ever smokers and *p*<0.001 for current smokers). Studies with no adjustments for cardiovascular risk factors found no significant association between smoking and risk of VTE, while the analysis of studies adjusted for cardiovascular risk factors, especially for BMI, yielded relatively higher RRs for ever and current smokers. There was evidence of moderate heterogeneity for former smokers (I^2^: 57.10% [95% CI 20.40%–76.88%], *p* = 0.01), but not for ever smokers (I^2^: 30.44% [95% CI 0%–60.69%], *p* = 0.11) or current smokers (I^2^: 19.73% [95% CI 0%–57.71%]). In a case-control study by Zhu et al. [43], the adjusted risk estimate for former smokers (RR: 3.25 [95% CI 1.92–5.49]) was much higher than the pooled risk estimate. After excluding this single study, there was no evidence of heterogeneity (I^2^: 0% [95% CI 0%–58.32%], *p* = 0.44) and the pooled risk estimate still reached statistical significance (RR: 1.09 [95% CI 1.05–1.12]) (Figure 4). The risk for developing VTE was significantly higher in current smokers than former smokers after adjustment for BMI (*p*<0.001).

**Table 4:** Stratified analysis of pooled relative risks of VTE for smokers and heterogeneity analysis^a^.

Factors Stratified	Ever Versus Never Smoker				Current Versus Never Smoker				Former Versus Never Smoker			
	Events	Individuals	RR (95% CI)	*p*-Value	Events	Individuals	RR (95% CI)	*p*-Value	Events	Individuals	RR (95% CI)	*p*-Value
**All studies**	35,151	3,966,184	1.17 (1.09–1.25)		22,991	2,729,153	1.23 (1.14–1.33)		23,911	2,759,336	1.10 (1.04–1.17)	
**Levels of adjustments** b												
−	1,492	3,645	1.23 (0.89–1.70)	0.03	129	258	0.50 (0.26–0.96)	<0.001	-	-	-	
+	1,309	34,055	0.90 (0.80–1.01)		319	6,100	1.21 (0.53–2.74)		223	3,971	1.04 (0.80–1.36)	0.72
++	32,350	3,928,484	1.21 (1.15–1.26)		22,543	2,722,795	1.30 (1.24–1.37)		23,688	2,755,365	1.10 (1.03–1.17)	
**Type of studies**												
Case-control	9,460	40,136	1.24 (1.07–1.44)	0.39	4,784	10,961	1.06 (0.82–1.38)	0.40	4,946	12,238	1.44 (1.06–1.95)	0.10
Cohort	25,691	3,926,048	1.14 (1.07–1.22)		18,207	2,718,192	1.26 (1.16–1.37)		18,965	2,747,098	1.07 (1.04–1.11)	
**Sex** c												
Men	10,190	1,248,414	1.17 (1.04–1.33)	0.84	6986	984,914	1.35 (1.21–1.50)	0.64	7,428	845,657	1.05 (0.99–1.11)	0.22
Women	20,179	2,520,580	1.18 (1.11–1.25)		14,751	1,861,473	1.30 (1.19–1.41)		15,698	1,887,885	1.10 (1.05–1.15)	
**Type of VTE** c												
Unprovoked	2,854	270,411	1.19 (1.08–1.30)	0.80	1,648	177,508	1.28 (1.16–1.42)	0.73	1,765	175,242	1.06 (0.95–1.18)	0.52
Provoked	2,461	138,705	1.16 (1.04–1.30)		1,504	92,497	1.32 (1.15–1.52)		1,842	80,316	1.08 (0.96–1.23)	
**Site of VTE** c												
DVT	5,252	1,185,571	1.21 (1.08–1.36)	0.98	4,709	839,355	1.39 (1.22–1.59)	0.99	4,496	928,758	1.10 (1.00–1.22)	0.88
PE	4,678	1,461,612	1.22 (1.09–1.37)		2,907	966,032	1.38 (1.22–1.56)		2,660	1,047,304	1.08 (0.90–1.30)	
**Smoking as the primary analysis**												
Yes	33,226	3,955,855	1.19 (1.12–1.26)	0.55	22,587	2,723,414	1.31 (1.24–1.37)	0.001	23,710	2,755,719	1.10 (1.04–1.17)	0.72
No	1,925	10,329	1.15 (0.90–1.47)		404	5,739	0.72 (0.44–1.17)		201	3,617	1.01 (0.77–1.34)	
**VTE validation**												
Yes	11,384	258,379	1.20 (1.07–1.34)	0.68	6,135	85,621	1.16 (1.00–1.36)	0.46	6,114	78,665	1.17 (1.03–1.34)	0.27
No	23,767	3,707,805	1.16 (1.08–1.25)		16,856	2,643,532	1.31 (1.22–1.40)		17,797	2,680,671	1.07 (1.02–1.13)	
**Geographical area**												
Europe	27,671	3,638,051	1.20 (1.10–1.31)	0.33	18,634	2,587,558	1.29 (1.18–1.41)	0.18	18,917	2,609,422	1.08 (1.04–1.11)	0.39
North America	6,002	324,642	1.10 (0.99–1.23)		3,989	140,725	1.16 (1.01–1.35)		4,621	149,091	1.09 (1.00–1.20)	
Asia	1,478	3,491	1.29 (0.95–1.77)		368	870	0.87 (0.57–1.33)		373	823	3.25 (1.92–5.49)	
**Source of patients**												
Population based	17,443	1,626,679	1.15 (1.09–1.23)	0.39	11,425	1,042,493	1.27 (1.17–1.38)	0.03	11,702	1,128,036	1.10 (1.06–1.15)	0.002
Hospital based	2,594	6,125	1.30 (1.00–1.69)		497	1,128	0.70 (0.41–1.19)		373	823	3.25 (1.92–5.50)	
**Publication year**												
≤2,000	688	242,655	1.35 (0.89–2.05)	0.61	246	80,207	1.48 (1.04–2.11)	0.40	222	83,114	0.95 (0.71–1.27)	0.53
>2,000	34,463	3,723,529	1.16 (1.09–1.24)		22,745	2,648,946	1.22 (1.13–1.33)		23,689	2,676,222	1.10 (1.04–1.17)	

a *p*-Values test homogeneity between strata.

b Levels of adjustment in multivariate models: −, not adjusted for any confounding factors; +, adjusted for conventional confounding factors (i.e., age, sex); ++, further adjusted by potential cardiovascular risk factors (BCDHAP: B, BMI, body weight, circumference; C, cholesterol; D, diabetes; H, hypertension; A, alcohol consumption; P, physical activity. BMI, body weight or circumference was adjusted in every study).

c Studies could contribute to one or both estimates depending on design of the primary studies.

We undertook meta-regression to further identify the relationship between BMI and smoking-VTE risk. Although baseline BMI did not seem to be significantly correlated with the smoking-VTE risk for ever smokers, BMI-adjusted risk estimates were significantly higher than unadjusted ones (*p* = 0.02) (Figure S6).

We also evaluated whether a difference existed between men and women, DVT and PE, and unprovoked and provoked VTE in the smoking-VTE relationship. The stratified analyses shown in Table 4 suggest no modification of the relationship by these characteristics. To allow an unbiased comparison, we also calculated the RRs from studies reporting both estimates for men and women, DVT and PE, and unprovoked and provoked VTE. Similar pooled risks were again observed in both sexes (*p* = 0.95) [7],[8],[26],[37], sites of VTE (*p* = 0.31) [29],[33],[37], and types of VTE (*p* = 0.38) [8],[22],[27],[28],[33],[38],[39],[48].

### Dose-Response Relationship and Incidence of VTE

**Figure 5:**
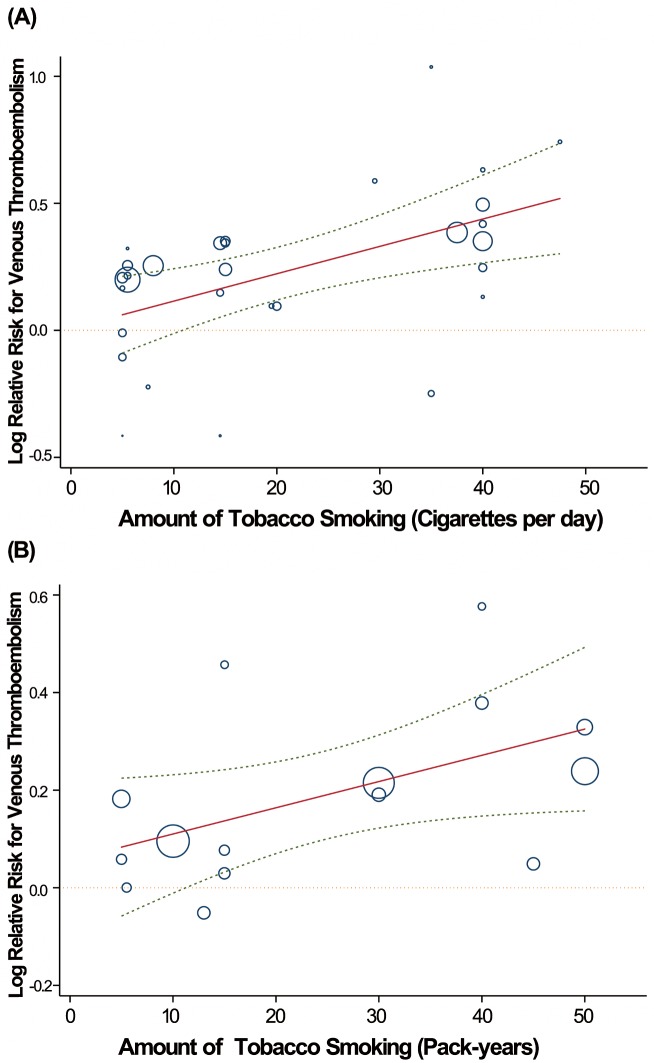
Linear dose-response relationship between relative risk of VTE incidence and tobacco consumption with cigarettes per day (A) and pack-years (B) as the explanatory variables. The solid line represents point estimates of association between tobacco consumption and VTE risk; dashed lines are 95% CIs. Circles present the dose-specific RR estimates reported in each study. The area of each circle is proportional to the inverse variance of the RR. The dotted line represents the null hypothesis of no association. The vertical axis is on a log scale.

After evaluating dose-response patterns for cigarettes per day and pack-years for ever versus never smokers, we observed a linear increase in VTE risk with increasing smoking consumption. The risk increased by 10.2% (95% CI 8.6%–11.8%) for every additional ten cigarettes per day or by 6.1% (95% CI 3.8%–8.5%) for every additional ten pack-years (for example, an individual who smoked one pack of cigarettes per day for 40 y or two packs per day for 20 y has a relative increased risk of 26.7% [95% CI 16.0%–38.4%] for developing VTE compared with someone who never smoked) (Figure 5).

From eight population-based studies that reported information on person-years in smokers and nonsmokers, we could calculate absolute annual rates of VTE cases from the general population: 176.3 cases per 100,000 person-years in smokers and 152.0 cases in nonsmokers, corresponding to an absolute risk increase of 24.3 (95% CI 15.4–26.7) cases per 100,000 person-years.

### PAF Calculations

Using the average prevalence of smoking from included cohort studies and the summary estimates obtained from all studies combined, the PAF of VTE due to smoking were 8.7% (95% CI 4.8%–12.3%) for ever smoking, 5.8% (95% CI 3.6%–8.2%) for current smoking, and 2.7% (95% CI 0.8%–4.5%) for former smoking. If cardiovascular risk factor-adjusted risk estimates were used, then the proportions of VTE explained by three categories of smoking increased to 10.6% (95% CI 7.8%–12.8%), 7.7% (95% CI 6.1%–9.1%), and 2.8% (95% CI 1.1%–4.5%), respectively.

## Discussion

The present meta-analysis, involving approximately 4 million participants and more than 35,000 patients with VTE from 32 observational studies, found a slightly increased risk of VTE for smokers compared with non-smokers. The risk was higher in studies adjusted for conventional cardiovascular risk factors, especially for BMI. The risk of developing VTE was greater for current smokers than for former smokers, and a dose-response relationship was found for daily smoking and pack-years smoked.

Recent studies have suggested that patients with obesity, hypertension, diabetes, or dyslipidemia were at risk of developing VTE, whereas conflicting results were reported for smoking [10],[11],[49]–[53]. This meta-analysis is the first to our knowledge to confirm smoking to be an independent risk factor for VTE. The risk magnitude appears to be less robust than those reported for well-established major risk factors such as cancer, surgery, pregnancy, use of estrogens, or mutation of factor V Leiden and prothrombin [54]–[57]. However, smoking is more common and its coexistence is associated with an additive causative effect. For example, there was a synergistic effect on VTE risk for smoking and oral contraceptive use. Pomp et al. reported the OR of developing VTE for oral contraceptive users was 3.90, but increased to 8.79 when current smoking was added [37]. One prospective cohort study also identified a hazard ratio of 3.75 for the association of the combination of current smoking and the prothrombin mutation with the risk of VTE, significantly higher than that of the prothrombin mutation only [58]. Thus, given the multi-factorial nature of VTE, it is highly likely that the concomitant action of smoking may be responsible for a proportion of VTE in the general population.

A causal relationship between VTE and smoking may be mediated by different mechanisms. Our results suggest that the association of smoking with VTE risk may be largely mediated by an acute mechanism, supported by a dose-response relationship for the amount of current smoking and the higher risk in current compared to former smokers. In addition, the association was not solely due to smoking-related secondary diseases, because we found a positive association between current smoking and both unprovoked and provoked VTE. Furthermore, there is biological plausibility for the relationship. A procoagulant state, reduced fibrinolysis, inflammation, and increased blood viscosity may underlie the association between smoking and VTE risk [59]–[61]. Smoking is associated with a higher level of plasma fibrinogen, hence the increase of factor VIII, which has been reported to be associated with VTE [62]. It has been shown that the fibrinogen concentration decreased quickly after cessation of smoking and the fibrinogen concentration was nearly equal in former smokers and never smokers [63],[64]. Yarnell et al. detected a positive relationship between the amount of current tobacco consumption and plasminogen activator inhibitor-1 concentration, which may also be related to VTE [65]–[67]. These findings might suggest an acute causal association and the dose-response relationship between VTE and smoking. However, a relatively weak association between former smoking and the risk of VTE was also observed. We suppose this association may be mediated by secondary smoking-related diseases. Former smoking is related to cardiovascular diseases, diabetes, and certain types of cancer [68]–[70], which may be associated with risk of VTE [54],[71]. It is also possible that chronic inhalation of tobacco smoke, causing progressive lung destruction, chronic obstructive pulmonary disease, and emphysema [72], may also result in a hypercoagulable state and thus contribute to an increased risk of VTE [73].

It is of note that lack of adjustment for BMI tends to deflate the pooled risk estimate, indicating that BMI is an important confounding factor when assessing the smoking-VTE association. Limiting studies to those adjusted for BMI identified no significant heterogeneity for ever and current smokers, suggesting that BMI may be a source of heterogeneity. Current smokers tend to be thinner than nonsmokers or former smokers [74]–[76], and several studies have shown that smokers' BMI is lower [77]. However, previous studies also identified obesity or weight gain to be an independent risk factor for developing VTE [11],[20],[78]. Thus, given that body leanness of some smokers might partly reduce the risk, the true magnitude of association between smoking and VTE may be greater. This may be an explanation for the non-significant association observed in previous observational studies and meta-analysis that did not control for BMI.

Strengths of this meta-analysis include the strict inclusion criteria, the large number of patients analyzed, the robustness of the findings in sensitivity analyses, the dose-response relationship, and the fact that all subgroup analyses were prespecified a priori. The absence of important publication bias supports the robustness of the study findings. A possible limitation of our study is the heterogeneity of the studies with regard to adjustment of the estimates for potential confounders. Although differences in levels of adjustment seems, at least in part, to explain this finding, heterogeneity still exists in former smokers, even after we confined the analysis to studies that adjusted for BMI. This suggests that apart from BMI, there are other factors that potentially may confound the risk estimates. Furthermore, baseline BMI was not significantly associated with the smoking-VTE risk, indicating that the relation between BMI and risk of VTE in smokers needs to be further elucidated. Inclusion of different types of studies into one meta-analysis may also introduce heterogeneity into the results. Despite this, the consistency of the finding of an increased risk of VTE among smokers in both case-control and cohort studies suggests that the association is valid. In addition, the results from a given study for the three comparisons (ever versus never, former versus never, current versus never) are not independent and there is a possibility of a type I error. However, the results continued to be statistically significant after adjusting for multiple comparisons by setting α = 0.05/3. Like all meta-analyses, our study has the limitation of being a retrospective analysis. Another limitation was the lack of individual participant data, which precluded determining the independent associations of individual variables with study outcomes. Instead, we used between-study meta-regressions, when possible.

In conclusion, the results from this meta-analysis suggest that smoking slightly increases the risk of VTE, independent of conventional cardiovascular risk factors. BMI may be a potential confounding factor in the risk estimates. The association between smoking and VTE has clinical relevance with respect to individual screening, risk factor modification, and the primary and secondary prevention of VTE. Future prospective studies are needed to elucidate the specific pathogenic mechanisms.

## Supporting Information

Figure S1 **Forest plot for VTE incidence: risk estimates for current versus never smokers.** The size of each square is proportional to the study's weight (inverse of variance).
(TIF)

Figure S2 **Forest plot for VTE incidence: risk estimates for former versus never smokers.** The size of each square is proportional to the study's weight (inverse of variance).
(TIF)

Figure S3 **Pooled relative risks of VTE for ever smokers stratified by VTE validation.** VTE case confirmation was based on medical record, radiology, or autopsy (validated) and questionnaire or patient registry (not validated).
(TIF)

Figure S4 **Pooled relative risks of VTE for current smokers stratified by VTE validation.** VTE case confirmation was based on medical record, radiology, or autopsy (validated) and questionnaire or patient registry (not validated).
(TIF)

Figure S5 **Pooled relative risks of VTE for former smokers stratified by VTE validation.** VTE case confirmation was based on medical record, radiology, or autopsy (validated) and questionnaire or patient registry (not validated).
(TIF)

Figure S6 **Relationship between baseline BMI and smoking-VTE risk for ever smokers.** Regression analyses were stratified, where appropriate, by level of adjustment for BMI. Meta-regression *p* = 0.64 for BMI-unadjusted risk estimates, *p* = 0.92 for BMI-adjusted risk estimates.
(TIF)

Text S1 **PRISMA 2009 checklist.**
(DOC)

## Abbreviations

BMI: body mass index
DVT: deep vein thromboembolism
OR: odds ratio
PE: pulmonary embolism
RR: relative risk
VTE: venous thromboembolism
