# Supplementary figures and images for: Current and Former Smoking and Risk for Venous Thromboembolism: A Systematic Review and Meta-Analysis
Source: PLoS Med. 2013 Sep 17;10(9):e1001515. doi: 10.1371/journal.pmed.1001515 (PMC3775725; doi:10.1371/journal.pmed.1001515)

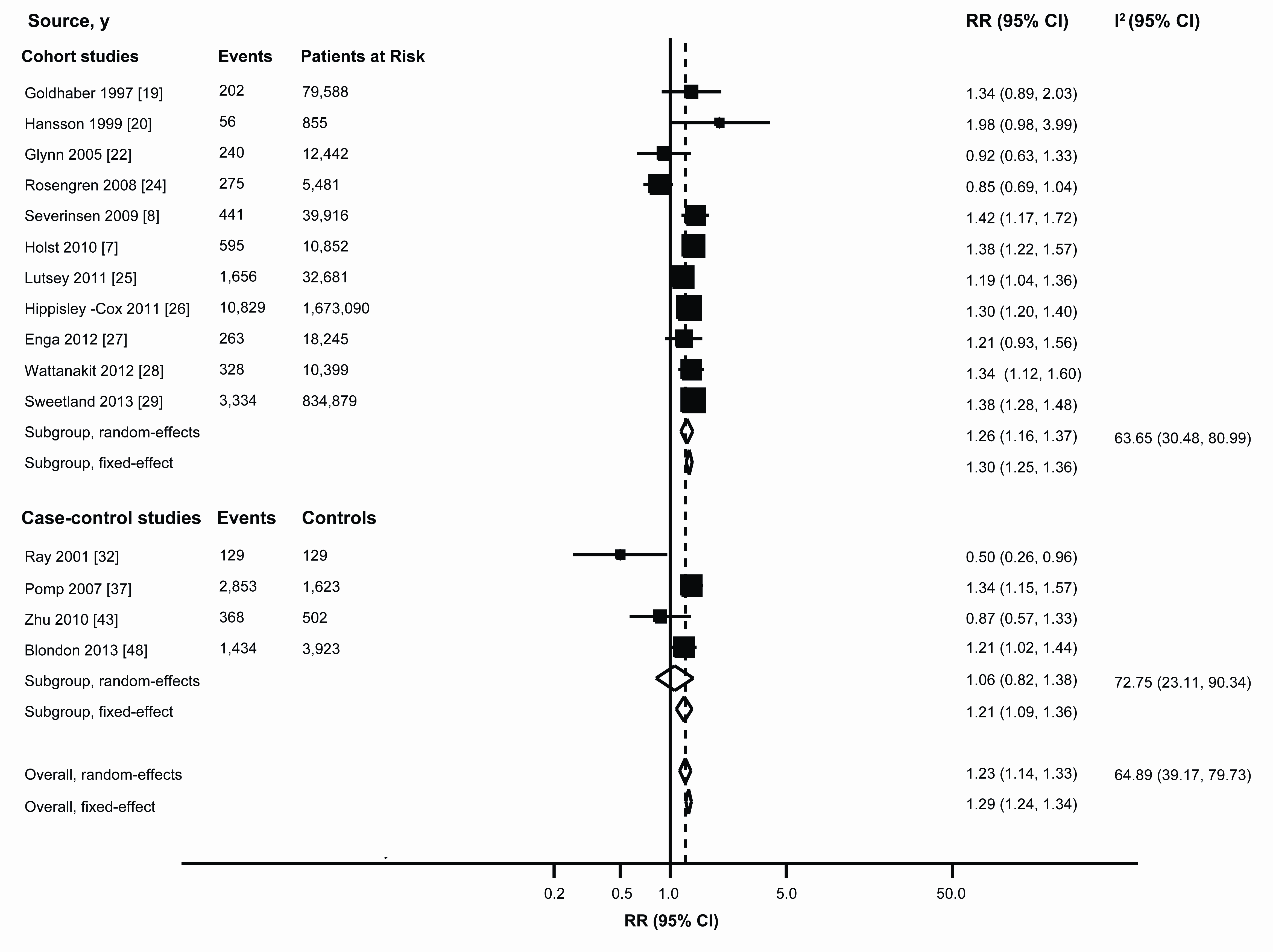

Supplement: Figure S1 — Forest plot for VTE incidence: risk estimates for current versus never smokers. The size of each square is proportional to the study's weight (inverse of variance). (TIF) [file pmed.1001515.s001.tif]

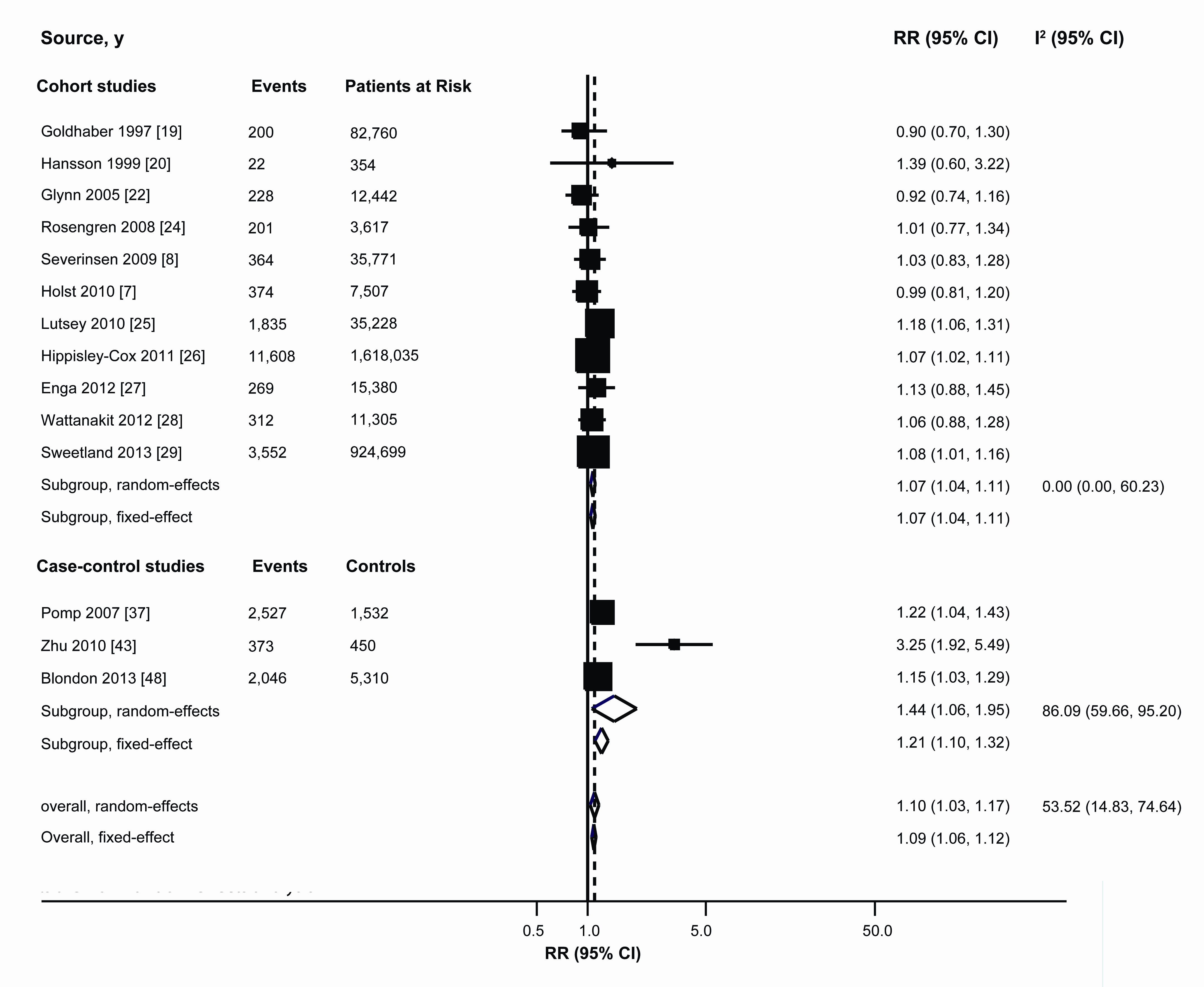

Supplement: Figure S2 — Forest plot for VTE incidence: risk estimates for former versus never smokers. The size of each square is proportional to the study's weight (inverse of variance). (TIF) [file pmed.1001515.s002.tif]

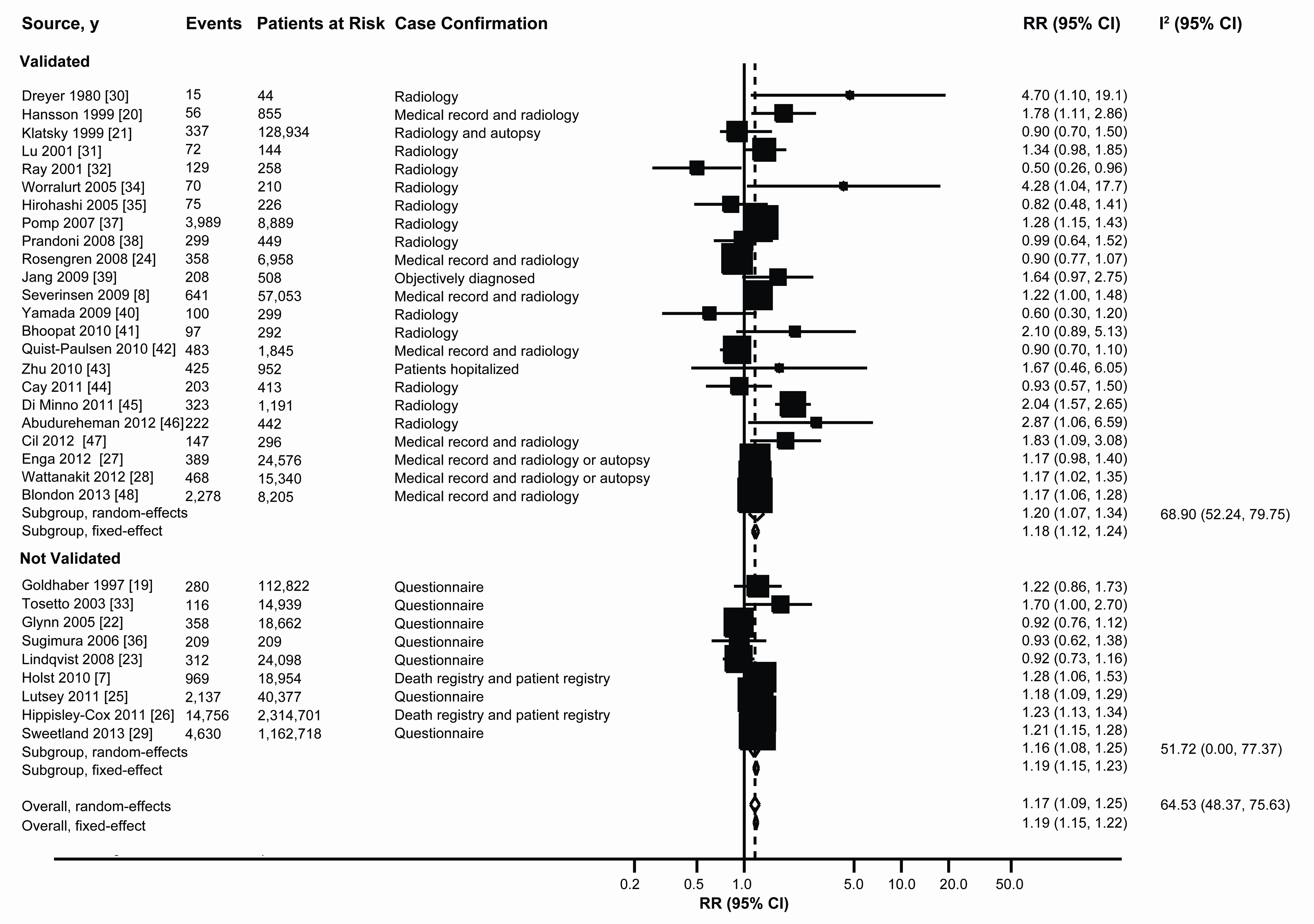

Supplement: Figure S3 — Pooled relative risks of VTE for ever smokers stratified by VTE validation. VTE case confirmation was based on medical record, radiology, or autopsy (validated) and questionnaire or patient registry (not validated). (TIF) [file pmed.1001515.s003.tif]

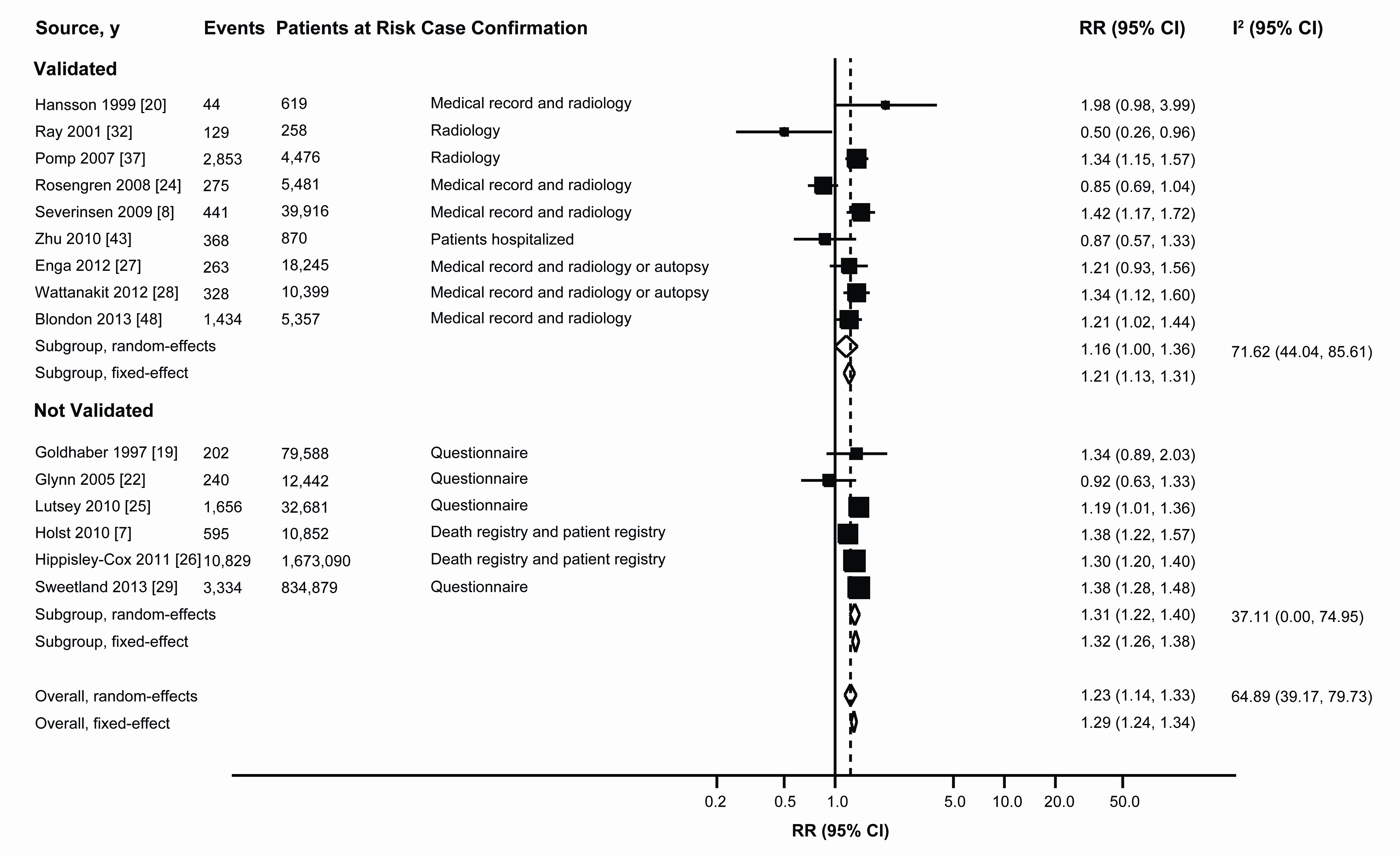

Supplement: Figure S4 — Pooled relative risks of VTE for current smokers stratified by VTE validation. VTE case confirmation was based on medical record, radiology, or autopsy (validated) and questionnaire or patient registry (not validated). (TIF) [file pmed.1001515.s004.tif]

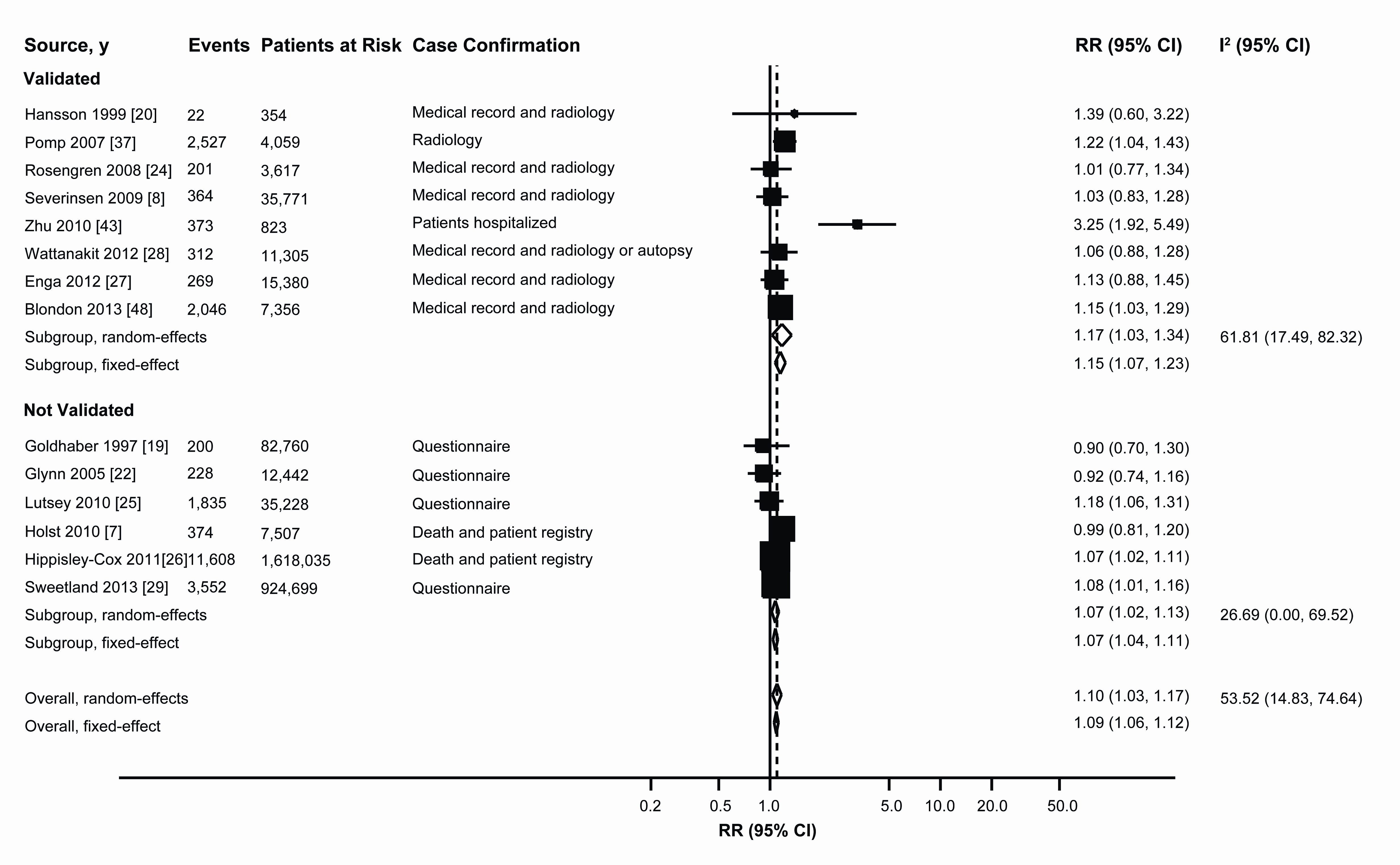

Supplement: Figure S5 — Pooled relative risks of VTE for former smokers stratified by VTE validation. VTE case confirmation was based on medical record, radiology, or autopsy (validated) and questionnaire or patient registry (not validated). (TIF) [file pmed.1001515.s005.tif]

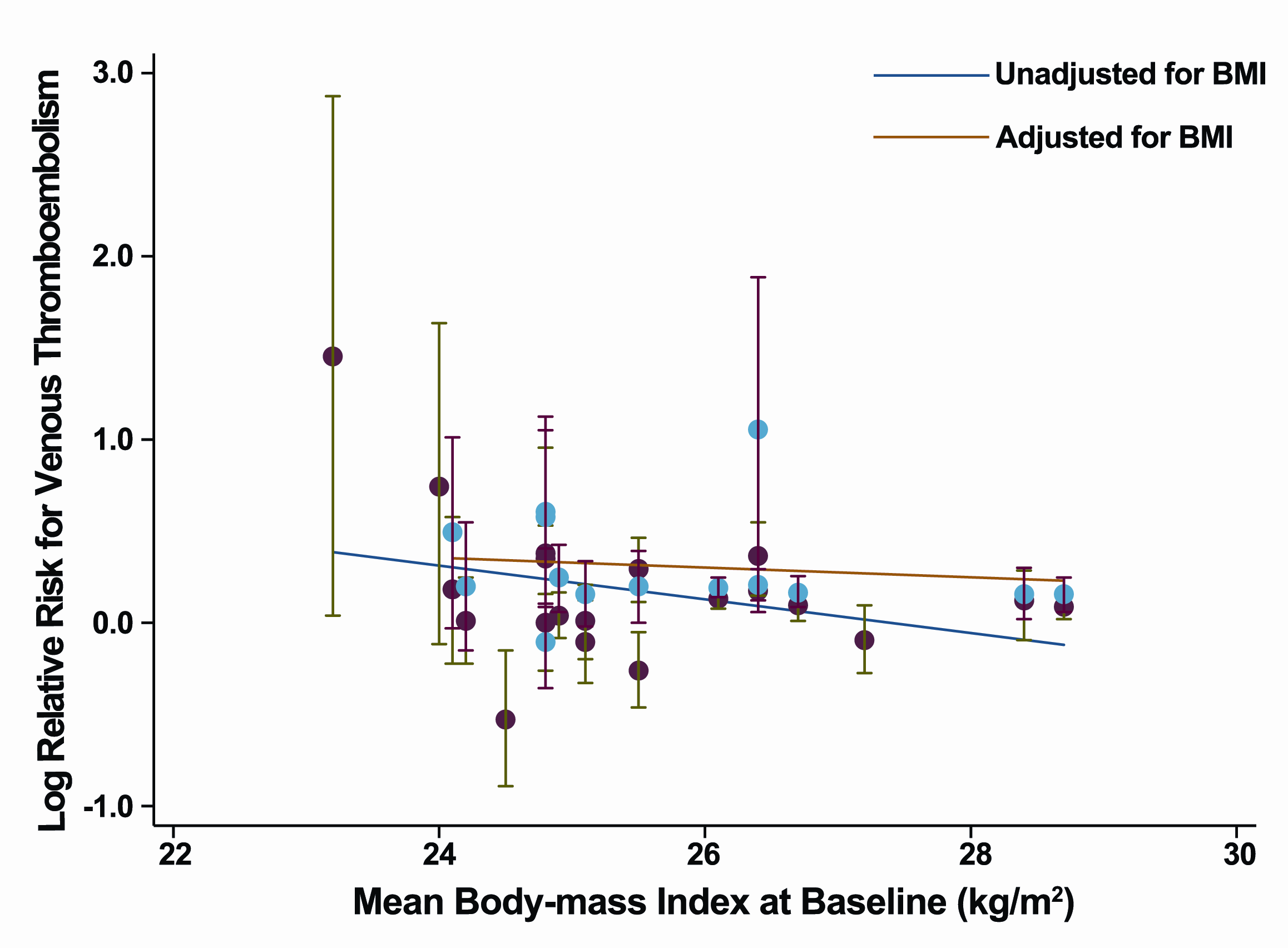

Supplement: Figure S6 — Relationship between baseline BMI and smoking-VTE risk for ever smokers. Regression analyses were stratified, where appropriate, by level of adjustment for BMI. Meta-regression p = 0.64 for BMI-unadjusted risk estimates, p = 0.92 for BMI-adjusted risk estimates. (TIF) [file pmed.1001515.s006.tif]
